# A quantitative approach to understanding the effect of the COVID-19 pandemic on training opportunities for neurosurgical trainees in England

**DOI:** 10.1308/rcsann.2025.0090

**Published:** 2026-01-20

**Authors:** D Thompson, A Williams, D MacArthur, S Thomson, A Helmy

**Affiliations:** ^1^ University of Cambridge, UK; ^2^ North Bristol NHS Trust, UK; ^3^ Nottingham University Hospitals NHS Trust, UK; ^4^ E-logbook, an intercollegiate project of the Royal Colleges of Surgeons of UK and Ireland, UK

**Keywords:** COVID-19, Neurosurgical training, Quantitative analysis

## Abstract

**Introduction:**

The literature speaks to the impact of the COVID-19 pandemic having a profound effect on surgical training. Our objective in this study was to quantify the effect of the COVID-19 pandemic on neurosurgical training and to test whether an effect on the quality of neurosurgical training can be inferred from a quantitative methodology.

**Methods:**

Surgical training episodes logged by neurosurgical trainees with a National Training Number were provided by e-logbook for the period January 2019 to December 2023. This was crosslinked with trainee data provided by the Intercollegiate Surgical Curriculum Programme and compared with data from the Capse Healthcare Knowledge System, which records operative spells in English neurosurgical units, over the same period.

**Results:**

Some 24,416 surgical training episodes were logged by trainees in 2023 compared with 32,033 in 2019. The ratio of surgical training episodes logged to operative spells recorded increased from 0.74 to 0.84 between 2019 and 2021, but fell to 0.72 by 2023. When filtered for elective cranial surgical training episodes logged compared with operative spells, the data show a significant drop from 67% to 60%. However, spinal surgical training episodes logged have risen from 58% to 70% of operative spells, although the number of surgical training episodes logged has declined by 1,118. The average number of surgical training episodes logged per year per trainee in 2019–2020 was 132, and this has risen every year and stands at 173 in 2022–2023.

**Conclusions:**

The primary findings of this study are that the recording of training events is below pre-pandemic levels. In total, 4,617 fewer cases were logged in 2023 than in 2019 and the proportion of elective cranial cases logged compared with operative spells fell from 67% in 2019 to 60% in 2023. This study suggests further efforts are needed to safeguard training opportunities and maintain a high quality of training.

## Introduction

The COVID-19 pandemic had a significant effect on the operational behaviour of every surgical centre in the United Kingdom (UK), both in terms of the quantity of operations being performed and the surgical case-mix.^1^ We have previously demonstrated that neurosurgical emergency work was generally well maintained; however, there was a significant decline over the period in terms of elective work, especially with respect to spinal surgery cases.^2^ Neurosurgical units throughout England are generally still not performing numbers of operations in line with pre-pandemic levels.^2^ Trainees of all medical and surgical specialties were affected by the COVID-19 pandemic through redeployment, illness, stress and the challenges facing a healthcare system with an ever increasing backlog of patients.^3-5^ Training programmes have attempted to tackle the disruption and the Department of Health and Social Care has awarded funding to “training recovery programme interventions” that aim to “address the impact of the pandemic on curriculum attainment, progression, and wellbeing”.^6^ Questions remain in neurosurgery with respect to how significant the initial impact was on training and whether initiatives to address this impact have been successful. We sought to provide a quantitative analysis of training opportunities between 2019 and 2023 to provide insights that can inform neurosurgical training.

The availability of big data resources and means for analysing these data can provide surgical specialties with unique insights into trainee exposure to operating during training.^7^ Although survey data on the impact of the COVID-19 pandemic on medical training are readily available, a quantitative analysis as to the precise impact of the COVID-19 pandemic on neurosurgical training has not been performed. We hypothesised that the pandemic would have had an effect on the quantity of surgical training episodes that neurosurgical trainees are logging, as well as the quality of their training, with respect to both the type of supervision and the range of exposure to different subspecialties. Disruption to surgical training in general has been of significant concern in the literature.^8-10^ The recovery phase has given rise to novel means of increasing patient throughput, such as surgical hubs and greater work in independent sector providers, which may further decrease the pool of operations available for training opportunities unless efforts are made to prevent this.^11^

The primary objective of this study is to compare surgical training episodes logged with overall operative spells recorded in neurosurgical centres. Secondary aims include assessing whether there has been a change in supervision levels since the pandemic and to assess whether the case-mix has changed in general and across different training grades. These data provide an objective measure of the impact the COVID-19 pandemic has had on neurosurgical training and provide evidence for the rational planning of a future training structure.

## Methods

### Study design

The study was a retrospective audit of all surgical training episodes logged by neurosurgical trainees in England between 1 January 2019 and 31 December 2023.

### Data sources

The data utilised in this study were extracted from e-logbook with respect to surgical training episodes logged by trainees and data fields describing the timing, type and location of the operation. Data with respect to trainees and their training grade were crosslinked to the data set from information provided by the Intercollegiate Surgical Curriculum Programme (ISCP). This research used data assets made available as part of the ISCP and e-logbook databases supported by the Joint Committee on Surgical Training (JCST). The data were released following review of the application by the Data, Analysis, Audit and Research Group (DAARG). Extraction of the data sets was supported by the ISCP and e-logbook via the ISCP data manager and e-logbook data manager. All data management and analysis was undertaken by the independent research team. This paper presents independent research. The views expressed are those of the authors and not necessarily those of the ISCP, e-logbook or JCST. Data with respect to overall operative numbers in neurosurgery in England were compiled using CHKS Ltd’s iCompare platform. CHKS’s iCompare is an audit and benchmarking tool that allows for comparison of a bespoke peer group over a range of metrics.^12^ CHKS iCompare uses data provided by patients and collected by the National Health Service (NHS) as part of their care and support. Where Health Episode Statistics data are used, it is with permission of NHS England.

### Study population

All surgical training episodes where operations were coded as neurosurgical operations performed by neurosurgical trainees with a National Training Number (NTN) in England were included in the study. Operations logged by neurosurgical trainees with codes referencing other surgical specialties were not included. Overall operative output included inpatient spells for all patients of any age admitted during the period of study who underwent either a primary or secondary neurosurgical procedure in a neurosciences centre in England.

### Data wrangling

Data from the e-logbook platform coding all procedures logged by NTN neurosurgical trainees in England were merged with an Excel file describing the operation codes used to denote what surgery has been performed. This was subsequently merged with an extract from the ISCP platform that provided a pseudo-anonymised training identification (ID), the specialist year of the trainee, as well as the dates between which they were of that designation. The pseudo-anonymised training ID was used to merge these two files. Fields of operation date, operation code, operation description, operation type, operation category, training level, patient age, Confidential Enquiry into Perioperartive deaths (CEPOD) description and supervision level were contained in the overall file and used to provide other data outputs.

Surgical training episodes are defined by operative cases logged by trainees, whereas opportunity is defined by operative spells at adult neurosurgical centres that are recorded.

### Statistical analysis

Crude numbers of surgical training episodes logged by trainees were compared as a ratio against the number of operative spells recorded for neurosurgical patients admitted to the 24 adult neurosurgical centres in England during the same period. Operative spells are an underestimate of operation numbers because a patient can have more than one neurosurgical operation in the same spell, but these were the data available for comparison and the difference was consistent across all years and centres. Surgical training episodes per training year per trainee were assessed using a different period because training years run from August to August. If calendar years had been used for this analysis then it would overestimate the number of trainees in each year and therefore decrease the number of cases per trainee erroneously. A Pearson’s chi-squared test of independence was used to test whether the supervision levels had changed since the COVID-19 pandemic, which might be indicative of a qualitative change in neurosurgical training. Spearman’s rho correlation calculation was also performed to test whether there was monotonicity to the change, which would mean that surgical exposure was tending towards greater or lesser independence.

Data analysis was performed using Stata, version 17 (StataCorp LP, College Station, TX, USA).

## Results

During the period from 1 January 2019 31 December 2023 there were 145,823 surgical training episodes logged on e-logbook by neurosurgical trainees in England with NTN. Of these, 93,918 surgical training episodes were cranial, 42,745 were spinal operations and the remaining 9,165 were classified as ‘other’. During the same period there were 187,923 operative spells for patients admitted to the 24 adult neurosurgical centres in England.

### Comparison between surgical training episode numbers and operative spells

**Figure 1:**
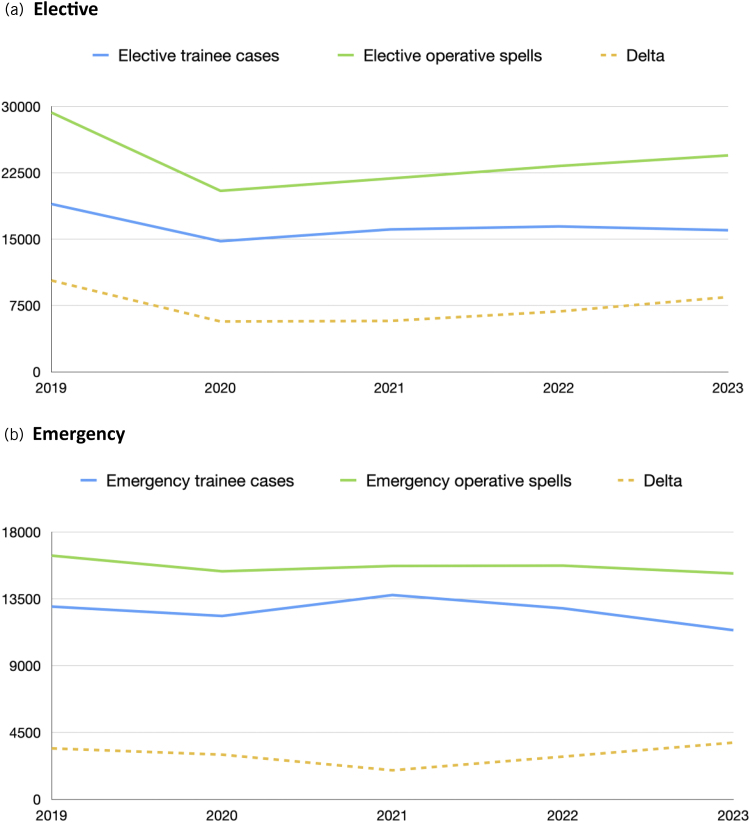
Number of elective (b) and emergency (b) cases logged by National Training Number neurosurgical trainees vs operative neurosurgical spells per year. The delta line represents the difference in number between operative spells and trainee cases logged.

**Figure 2:**
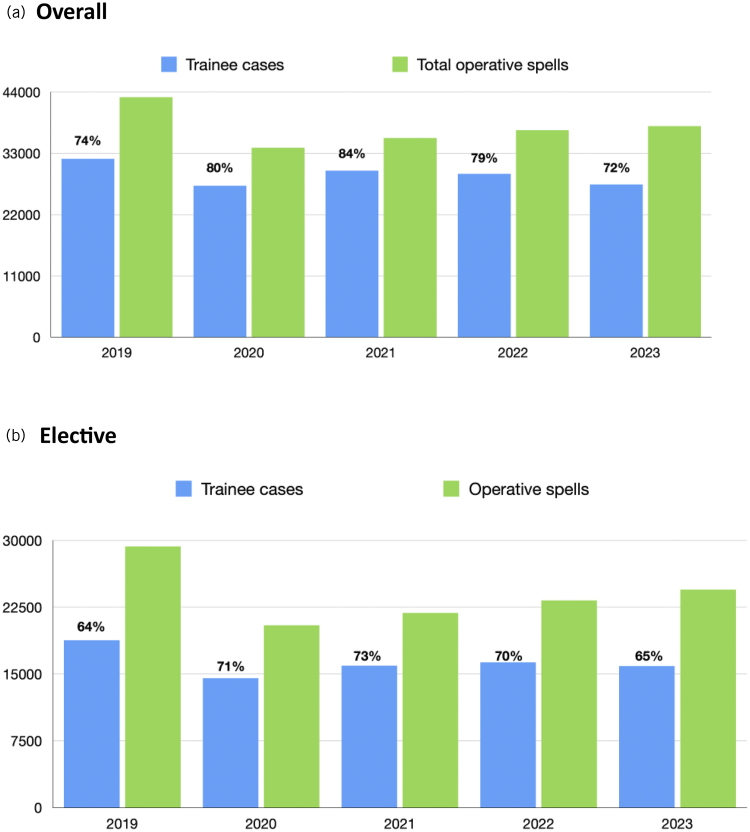
Overall (a) and elective (b) surgical training episodes logged as a proportion of operative spells recorded per year

**Figure 3:**
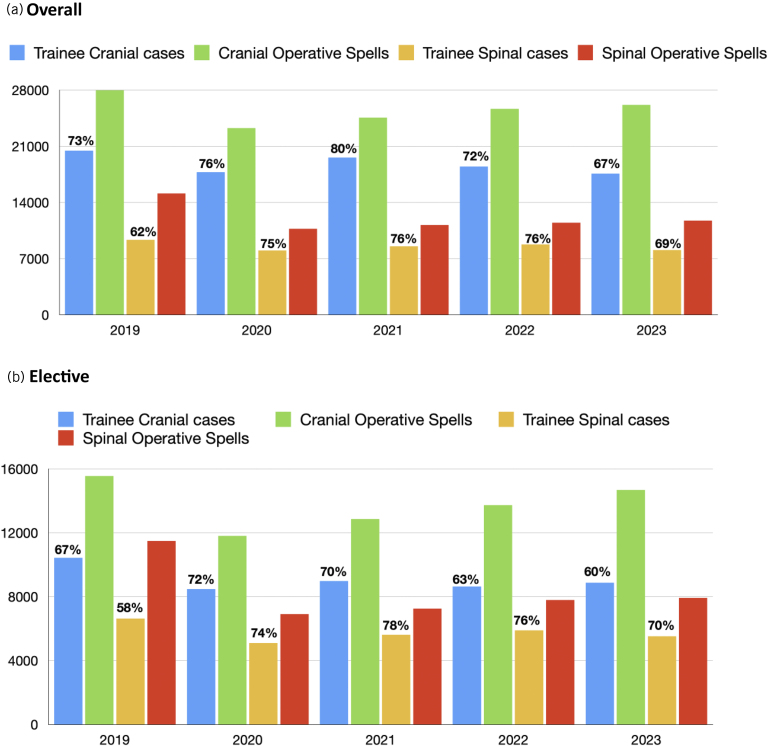
Overall (a) and elective (b) surgical training episodes as a proportion of operative spells defined as cranial or spinal per year

A time trend of logged surgical training episodes is presented in Appendix 1 (available online). A significant drop in the number of logged surgical training episodes is demonstrated following lockdown for the COVID-19 pandemic in March 2020; however, these numbers rebounded to be above January 2019 rates by September 2020. The year 2023 shows logged surgical training episodes to be generally below 2019 rates. If the data are subdivided into emergency and elective surgical training episodes logged and compared against neurosurgical operative spells recorded, it demonstrates that the difference between surgical training episodes logged and operative spells narrows for elective cases during the COVID-19 period before widening again in 2023 (Figure 1). For emergency cases there is a similar trend, with a narrowing of the gap in 2021 before widening again in 2023 to just above the difference in 2019. This trend is depicted again in Figures 2 and 3 and represented as surgical training episodes as a proportion of operative spells in Figure 2 and subdivided into cranial and spinal cases in Figure 3. The total number of operative spells in 2023 was 37,904 compared with 43,099 in 2019. The proportion of surgical training episodes to operative spells dropped to 72% between these periods, and in fact 4,617 fewer operations were logged in 2023 than in 2019. When filtered for elective cranial surgical training episodes logged compared with operative spells the data show a significant drop from 67% to 60%. However, spinal surgical training episodes logged has risen from 58% to 70% of overall operative spells, although the number of surgical training episodes logged has declined by 1,118.

### Supervision levels over time

**Figure 4:**
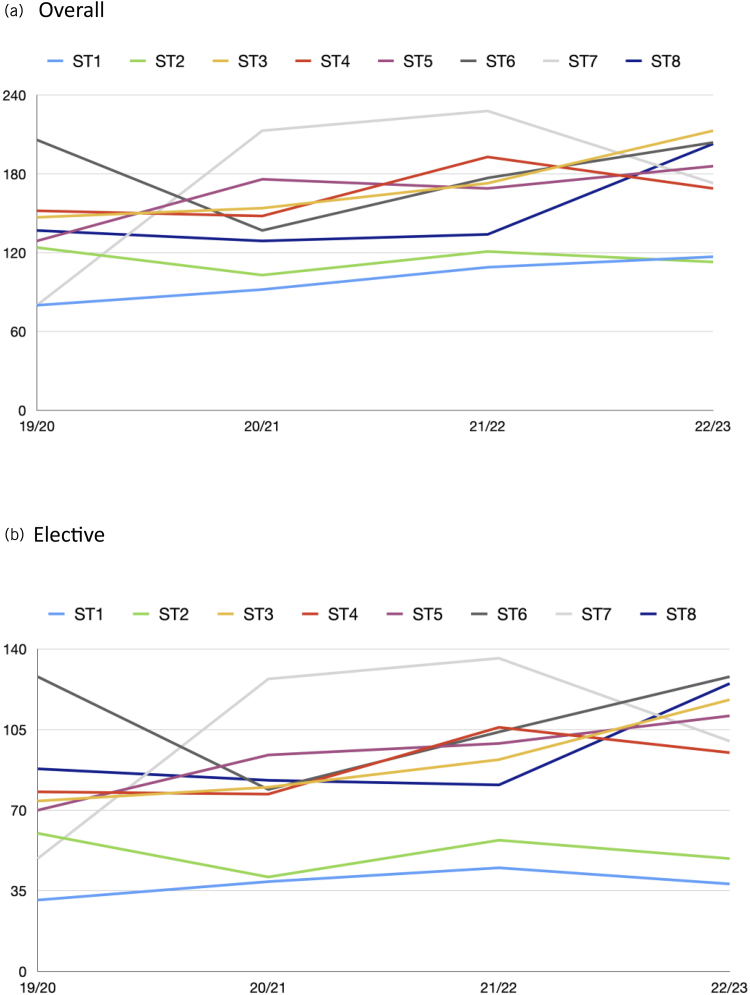
Overall (a) and elective (b) surgical training episodes logged per training grade per year

Supervision levels for surgical training episodes were tested against year using a Pearson’s chi-squared test of independence, which suggested that year and operative supervision levels were not independent (Table 1). Therefore a Spearman’s rho correlation calculation was performed, which measures the strength and directionality of the relationship of two variables. Spearman’s rho was −0.0099, which suggests that year and supervision are independent. Importantly, this demonstrates a lack of monotonicity. Supervision levels over time are depicted graphically in Appendix 2 (available online) and Figure 4. There is a shift during 2021 and 2022 to a greater number of supervised emergency surgical training episodes than those performed by trainees themselves. However, this appears similar to the relationship in 2019 by 2023.

**Table 1:** Pearson’s chi-squared test of supervision levels for trainee cases against time and Spearman’s rho correlation calculation

Year	Performed	STU/STS	Assisting	Observed	Total
2019	8,294 [7,659.7]	13,331 [14,146.0]	7,871 [7,727.2]	2,301 [2,264.1]	31,797
2020	5,937 [6,458.1]	11,895 [11,927.0]	7,123 [6,515.0]	1,854 [1,909.0]	26,809
2021	6,485 [7,144.6]	13,511 [13,194.9]	7,125 [7,207.6]	2,178 [2,111.9]	29,659
2022	7,026 [7,016.0]	13,320 [12,957.3]	6,755 [7,077.8]	2,024 [2,073.9]	29,125
2023	6,740 [6,563.6]	12,290 [12,121.8]	6,275 [6,621.4]	1,942 [1,940.1]	27,247
TOTAL	3,4842	64,347	35,149	10,299	144,637

Pearson chi-squared (16) = 277.6722 *p* < 0.01. Expected values are given in square brackets.

Spearman’s rho = −0.0099 *p* = 0.0002 [year and supervision level are independent]

STS = supervised trainer scrubbed; STU = supervised trainer unscrubbed

### Quality of training

The surgical training episodes logged per training year per trainee are shown in Figure 4. There is a general fluctuation on a yearly basis. However, if ST1 and ST2 are excluded then the numbers of surgical training episodes logged per trainee are more clustered in 2023 than in 2019, and more surgical training episodes on average are being logged per trainee. The average number of surgical training episodes logged per year per trainee in 2019–2020 was 132, and this has risen every year and stands at 173 in 2022–2023 (Figure 4a). This general trend is also present when filtered for elective operations as shown in Figure 4(b).

**Figure 5:**
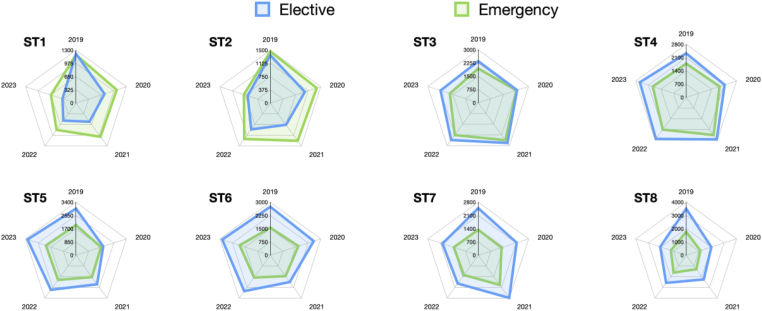
Distribution of surgical training episodes between elective and emergency procedures by trainee grade over time

The number of index cases logged – cases that trainees are mandated to perform a certain number of times during their training – are charted by supervision level against year in Appendix 3 (available online). If an index case is not logged as supervised trainer scrubbed/supervised trainer unscrubbed (STS/STU) or performed it does not ‘count’ towards the trainee’s overall index case number. This shows that the numbers and rates of supervision have remained stable over time. Figure 5 shows eight spider charts comparing elective and emergency surgical training episodes logged by each grade of trainee in each year. The traditional pattern of junior trainees having an even split of emergency to elective procedures, and senior trainees logging significantly more elective procedures, has changed, with the change being most pronounced for ST7- and ST8-level trainees.

Finally, the operative case-mix is demonstrated in Table 2, which breaks down the surgical training episodes logged by neurosurgical subspecialty. This shows that the proportion of cases logged for the various subspecialties has not significantly altered over the period in question.

**Table 2:** Case-mix of neurosurgical surgical training episodes logged by National Training Number neurosurgical trainees per year

	Number of training episode (%)												
Year	General and trauma	Functional	Epilepsy	Neuro-oncology	CSF	Skull base	Pituitary surgery	Neurovascular surgery	Cervical spine	Thoracic spine	Lumbar spine	Paediatrics	Other
2019	5,855 (18.1)	1,793 (5.5)	595 (1.8%)	5,084 (15.7)	5,743 (17.7)	573 (1.8)	710 (2.2)	1,230 (3.8)	2,408 (7.4)	385 (1.2)	4,310 (13.3)	3,083 (9.5)	660 (2.0)
2020	4,692 (17.1)	1,073 (3.9)	526 (1.9)	4,656 (17.0)	5,082 (18.5)	484 (1.8)	514 (1.9)	1,167 (4.3)	2,076 (7.6)	457 (1.7)	3,472 (12.7)	2,895 (10.6)	339 (1.2)
2021	5,408 (18.0)	1,381 (4.6)	547 (1.8)	4,681 (16)	5,370 (17.8)	547 (1.8)	596 (2.0)	1,318 (4.4)	2,055 (6.8)	456 (1.5)	3,912 (13.0)	3,438 (11.4)	411 (1.4)
2022	5,318 (18.1)	1,634 (5.6)	641 (2.2)	4,207 (14)	5,027 (17.2)	465 (1.6)	635 (2.2)	1,224 (4.2)	2,147 (7.3)	466 (1.6)	4,158 (14.2)	3,063 (10.5)	326 (1.1)
2023	4,729 (17.2)	1,524 (5.6)	608 (2.2)	4,147 (15)	4,874 (17.8)	528 (1.9)	680 (2.5)	1,224 (4.5)	1,995 (7.3)	469 (1.7)	3,948 (14.4)	2,267 (8.3)	425 (1.6)

CSF = cerebrospinal fluid procedures

## Discussion

The primary objective of this study was to assess whether the training of future UK neurosurgical consultants had been compromised by the impact of the COVID-19 pandemic on both surgical training episodes and aspects of service provision. Quantitatively we can see that the COVID-19 pandemic caused a significant drop in surgical training episodes logged, which coincided with lockdown.^13^ However, within 6 months these had returned to pre-pandemic rates. As previously described, the impact on emergency work was less dramatic and the number of surgical training episodes logged in 2020 (12,349) was similar to the number logged in 2019 (12,983).^2^ An interesting observation from the height of the COVID-19 pandemic (2020 and 2021) is that in these years a higher proportion of the available operations were made available to NTN neurosurgical trainees, a finding that now appears to have reversed. The difference between elective cranial surgical training episodes logged by trainees in 2023 compared with 2019 is stark and there are 1,555 fewer episodes, even though the operative spells have almost risen back to 2019 levels. The trend of a greater proportion of spinal surgical training episodes being logged by trainees needs to be seen in the context of significantly fewer opportunities being available, with 3,576 fewer operative spells in 2023 compared with 2019.

In terms of attempting to assess the quality of training opportunities using a quantitative approach, there is no statistically significant change in the level of supervision in the cases logged by trainees from greater to lesser independence. If cases were trending from performing to assisting then it could be hypothesised that pressures in theatres to deal with the large surgical backlog were affecting the exposure that consultants allow trainees during cases. However, we did not find evidence of this. In general, the case-mix of logged operations does not appear to have changed dramatically following the COVID-19 pandemic. The one subspecialty that does appear to have shown a decline is paediatric neurosurgery, which accounted for only 8.3% of the overall cases logged in 2023, with a peak of 11.4% in 2021. Although the direct effects of the COVID-19 pandemic were milder on children and young people, COVID had a profound effect on paediatric services and this continues to be a burden.^14^ The downturn in logged surgical training opportunities may reflect it being increasingly difficult for NTN neurosurgical trainees to gain appropriate paediatric neurosurgical exposure and trainers should be cognisant of this.

Overall surgical training episode numbers per trainee have not significantly deteriorated since 2019, and when divided into trainee grades we found growth in numbers per trainee comparing 2019–2020 with 2022–2023. There is some evidence that not all trainee grades were affected equally, however. ST1 and ST2 trainees appear to have struggled to maintain case numbers in the latter years, and especially in 2023. These numbers should not be falsely reassuring and are most likely to represent an attempt by trainees to compensate for the loss of opportunity in the years affected most directly by the COVID-19 pandemic. We would expect surgical training episodes per trainee per specialty training level to remain relatively static on a year-to-year basis in an ideal state, and therefore the significant fluctuation during this period is of concern. The fact that surgical training episodes per trainee logged in 2022–2023 have increased compared with 2019–2020 likely signifies training pressure rather than evidence of a good recovery of training opportunities.

The most notable finding from our study is that logged surgical training episodes are not in line with the numbers represented in 2019. Indeed, elective cranial logged training opportunities in particular appear to be well below the level expected if 2019 is taken as the pre-COVID index year. Furthermore, the significant narrowing of the gap between trainee-logged surgical training episodes and overall spinal operative spells speaks to a deficit in the latter in neurosurgical centres. It is concerning that logged surgical training episodes do not surpass those in 2019.

Furthermore, this study provides quantitative analysis to help us to answer the question of what good neurosurgical training looks like in the 21st century. The COVID-19 pandemic should not wholly distract from issues already present in neurosurgical training, such as redesigning training to respond to challenges posed by the European Working Time Directive and the contract for surgeons in training.^15^ Exact numbers of surgical training episodes needed by a trainee each year to demonstrate good surgical experience remains debatable. Indeed it is clear that although having a target of around 1,200 cases by the end of training makes the number of cases done a focus, measuring the quality of neurosurgical training is not a quantitative task. Figure 5 is perhaps the most instructive with respect to providing some insight into how the quality and type of training opportunities have been affected by the pandemic. The spider diagrams demonstrate well that in 2019 there was a progression from ST1 to ST8 with respect to the gap between emergency and elective surgical training episodes logged. ST1 and ST2 trainees require more emergency experience compared with elective as they develop generic neurosurgical skills and learn how to appropriately manage neurosurgical emergencies before their registrar years. The pattern through the registrar years is generally a greater split in favour of elective surgery towards ST8 because this is increasingly where the more meaningful training opportunities lie for increasingly skilled trainees. However, the years following 2019 show a deviation from this pattern and ST8-logged training opportunities include a higher proportion of emergency work than previously. The split of subspecialty surgical training episodes logged should also be of interest to the neurosurgery community. More than 50% of training opportunities are either spinal cases (17%), general and trauma cases (18%) and cerebrospinal fluid (CSF) cases (18%). Skull base, pituitary surgery and epilepsy surgery each represent under 3% of training opportunities. Each trainee’s training profile is unique as they seek to gain extra experience in their particular areas of interest; however, how much exposure a trainee should have as standard in the various subspecialties still requires attention.

We recognise that this is a quantitative analysis and looks at the picture of neurosurgical training from a national perspective. Our work on the recovery of operative spells following the COVID-19 pandemic demonstrated regional variation. Some deaneries were more operationally stressed than others and this likely leads to a widening of the gap in training opportunities between regions. The fact that case numbers logged by trainees are now lower than in 2019 suggests that there are some deaneries even further below these numbers, and this is of concern. This paper demonstrates the importance of utilising big data resources that are increasingly available to us to help audit neurosurgical practice, including training.^16^ Although it can only be an adjunct, this paper provides important information on the relative health of neurosurgical training. It is incumbent upon individual training programmes to ensure adequate training opportunities that are of sufficient quality to ensure that the neurosurgeons of tomorrow approach day one as a consultant with the requisite skillset. Hospitals currently face pressure from initiatives such as Getting It Right First Time (GIRFT) to maximise productivity using inspirational targets for theatre efficiency.^17,18^ What is not priced into the recovery plans is the effect on training, and policymakers need to consider the downstream effects of this. A radical solution might be to implement a lower tariff for operations that do not include surgical trainees. Although there is a risk of such a system being gamed, it would represent a commitment to ring-fencing surgical training opportunities that is currently lacking. The findings of this study should at least galvanise the neurosurgical community into considering how best we protect these opportunities in the face of the significant service pressures that have befallen the specialty in the post-COVID 19 era.

### Study limitations

There are several limitations to this study that should be addressed. First, the comparison of surgical training episodes logged by trainees and neurosurgical operative spells does not directly equate. Operative spells may include more than one neurosurgical operation. As well as this, more than one trainee may log the same operation as a surgical training episode. Patient hospital numbers were not available from the e-logbook data and therefore we cannot accurately detect whether a case has been logged twice by two separate trainees taking part in a single procedure. This is a systematic error throughout the paper, however; therefore we still find it a valid comparison of opportunity for surgical experience with cases logged. It also means that the gap between training opportunity and logged experience is larger than presented here.

Second, it must be stressed that the scope of this paper is primarily to assess the number of surgical training episodes logged by trainees. We cannot comment upon the quality of each training interaction. The number of surgical training episodes recorded by trainees does not speak to the overall quality of the training.

Third, as discussed previously, this is a national overview and therefore it may again provide false reassurance for some deaneries when in fact the gap has widened in terms of experience gained by trainees between deaneries.

Fourth, it should be highlighted that this paper covers only surgical training episodes logged by NTN neurosurgical trainees and does not include those cases logged by non-trainee registrars who make up a growing proportion of the workforce. A lower number of surgical training episodes logged by NTN neurosurgical trainees may speak to a greater number being logged by non-trainees.

Finally, this is predominantly a quantitative analysis and does not take account of additional hours that may have been worked; for example, to maintain training opportunities in line with 2019 numbers. It is important that this work be seen together with outputs such as the General Medical Council survey on trainee satisfaction to come to a better understanding of the state of neurosurgical training in England. Future work should look to better define what good training looks like as well as to gain a greater appreciation of the effect the shift of NHS cases into the independent sector is having on neurosurgical training.

## Conclusion

The primary findings of this study are that the recording of training events is below pre-pandemic levels. In total, 4,617 fewer cases were logged in 2023 than in 2019 and the proportion of elective cranial cases logged compared with operative spells fell from 67% in 2019 to 60% in 2023. Recorded supervision levels have not changed since the COVID-19 pandemic in a statistically significant way. The case-mix of subspecialty experience for trainees appears similar to 2019; however, paediatric cases are being logged at a lesser rate and this should be addressed by neurosurgical training programmes. This study suggests further efforts are needed to safeguard training opportunities and to ensure that these are of high quality.

## Competing interests

The authors have no conflicts of interest to declare.

## Funding

DT is the National Neurosurgical Audit Programme Fellow, a role wholly funded by the Society of British Neurological Surgeons. AH is supported by the Royal College of Surgeons of England and the Cambridge NIHR Biomedical Research Centre.

## Ethics approval and consent to participate

No patient identifiable data was used for the purposes of this study. Ethical approval was not required for this study in accordance with national guidelines.

## Artificial Intelligence

No generative artificial intelligence tools were used in the generation or editing of this manuscript, nor in data analysis or figure creation. Standard statistical software (Stata version 17; StataCorp, College Station, TX) was used for all analyses.

## Author contributions

DT conceived and designed the study, performed the data analysis, interpreted the findings, and drafted the manuscript. AW, AH, DM and ST contributed to study design, supervised analysis and interpretation, and critically revised the manuscript. All authors approved the final version of the manuscript.

## Supplementary Information

The online version contains supplementary material available at https://doi.org/10.1308/rcsann.2025.0090.
